# Hybridization ddRAD‐sequencing and phenotypic analysis clarify the phylogeographic structure and evolution of an alpine *Chrysanthemum* species with a sky island distribution

**DOI:** 10.3389/fpls.2025.1563127

**Published:** 2025-04-07

**Authors:** Xue-Ying Hu, Wen-Xun Lu, Zi-Zhao Wang, Guang-Yuan Rao

**Affiliations:** ^1^ State Key Laboratory of Protein and Plant Gene Research, School of Life Sciences, Peking University, Beijing, China; ^2^ Key Laboratory of Biodiversity Science and Ecological Engineering of the Ministry of Education, College of Life Sciences, Beijing Normal University, Beijing, China

**Keywords:** phylogeographic structure, speciation, sky island, hybridization ddRAD-sequencing, *Chrysanthemum hypargyrum*

## Abstract

The phylogeographic structure of a species is the result of historical intraspecific differentiation and influences the pace and trajectory of speciation. Therefore, study of the phylogeographic structure of species and the mechanisms underlying its formation can shed light on the evolutionary history and speciation of species, as well as enhance our understanding of the generation and maintenance of species diversity. *Chrysanthemum hypargyrum* is an alpine species endemic to central China. It is restrictively distributed to three isolated mountain ranges, and its populations exhibit a sky island distribution and some morphological variation to different environments. In this study, we investigated the morphogenetic divergence, phylogeographic structure, and evolutionary history of this species through hybridization ddRAD-sequencing, phenotypic analysis, and species distribution modeling. Our results indicate that *C. hypargyrum* originated in the Daba Mountains and has since diverged into three lineages. The phylogeographic structure and distribution of this species are mainly attributed to geographic isolation, the founder effect and Quaternary climate oscillations as its range expanded. The divergence of its three major lineages coincided with Pliocene mountain uplifts and Pleistocene climatic fluctuations. The current sky island distribution has also promoted the diversification and phylogeographic structure of *C. hypargyrum*.

## 1 Introduction

Speciation is a fundamental concept in evolutionary biology that has attracted considerable attention due to its implications for understanding the generation and maintenance of biodiversity (Ricklefs, 2006; Kopp, 2010; Schluter and Pennell, 2017). The current phylogeographic structure of a species is a product of historical intraspecific differentiation, and it affects the pace and trajectory of speciation (Avise et al., 1987; Avise and Walker, 1998; Leal et al., 2016). Therefore, study of the mechanisms underlying the phylogeographic structure of species can shed light on the process of speciation and facilitate the classification and conservation of species, especially for rare and endangered endemic species. An appropriate study system is required to address these issues.

*Chrysanthemum hypargyrum* Ling is an alpine species endemic to central China. Phylogenetic analyses of plastid and nuclear DNA sequences of *Chrysanthemum* species indicate that this species is a relatively early diverging lineage of this genus (Shen et al., 2021; Lu et al., 2022). Currently, *C. hypargyrum* only occurs in three isolated geographic regions, the Shennongjia region (SNJ), the Qinling Mountains (QLM), and the Hengduan Mountains (HDM), which correspond to three genetic lineages (Lu et al., 2022). Each lineage is distributed in high-altitude areas with very limited ranges. Therefore, *C. hypargyrum* exhibits a typical “sky island” distribution pattern. Sky island distributions, which occur in high-elevation areas isolated by inhospitable lowland habitats within mountain ranges, comprise unique ecosystems with distinctive biogeographical and ecological histories (McCormack et al., 2009; He and Jiang, 2014). Geographic isolation is the primary process underlying the evolution of sky island species in areas surrounded by low-elevation regions, which act as barriers to dispersal and facilitate the divergence of populations among sky islands (He and Jiang, 2014; Purushotham and Robin, 2016; Zhang et al., 2019; Atkins et al., 2020). Climatic oscillations can also influence species distributions within sky islands, which lead to range shifts during glacial and interglacial cycles (Hewitt, 1996; DeChaine and Martin, 2004; Galbreath et al., 2009; McCormack et al., 2009; Lu et al., 2022). Thus, the sky island distribution of *C. hypargyrum* provides an ideal setting for studying the roles of geography and ecology in speciation.

In addition, distinct patterns of differentiation in morphology and chromosomal ploidy have been observed among *C. hypargyrum* lineages based on our extensive field observations and a previous study (Lu et al., 2022). *C. hypargyrum* has long been thought to have yellow ray florets, and the floral color has actually differentiated within species. The Hengduan Mountains lineage has yellow ray florets, and the Shennongjia and Qinling lineages have white ones. Chromosomal ploidy has also diverged among lineages of this species; the Qinling lineage is tetraploid, and the Hengduan Mountains and Shennongjia lineages are diploid. Although earlier phylogenetic analyses resolved the phylogenetic placement of *C. hypargyrum* within the *C. zawadskii* complex (Shen et al., 2021; Lu et al., 2022), the phylogenetic relationships and demographic history between and within lineages have not been well studied due to a lack of sampling effort and appropriate molecular markers.

Here, we used morphological and hyRAD (hybridization ddRAD) data to investigate the phylogeographic structure and demographic history of this species. HyRAD combines the strengths of RAD and target enrichment, which makes it particularly suitable for studies of the phylogeographic structure of non-model species with large genomes (Suchan et al., 2016; Lang et al., 2020). Unlike traditional RAD methods, the success of hyRAD does not depend solely on the conservativeness of restriction enzyme cutting sites but also on the similarity between bait and target sequences (Suchan et al., 2016). Thus, hyRAD provides advantages for the retrieval of flanking sequences, which reduces missing data and enhances data homology (Andrews et al., 2016; Suchan et al., 2016). This method not only provides a large amount of data on genetic variation across the whole genome but also helps explain the phylogeography and demographic history of this species.

In this study, we aimed to clarify the evolutionary mechanisms that shaped the genetic differentiation and speciation of this alpine species by studying the phylogeographic patterns in the genetic and phenotypic characteristics of *C. hypargyrum*. We used data from hyRAD, morphological traits, chromosomal ploidy, and species distribution modeling to (1) identify the main geographical and ecological factors underlying the sky island distribution of *C. hypargyrum* and (2) determine how the sky island distribution of this species affects population genetic structure and the evolutionary history of populations.

## 2 Materials and methods

### 2.1 Taxon sampling and ploidy examination

**Figure 1:**
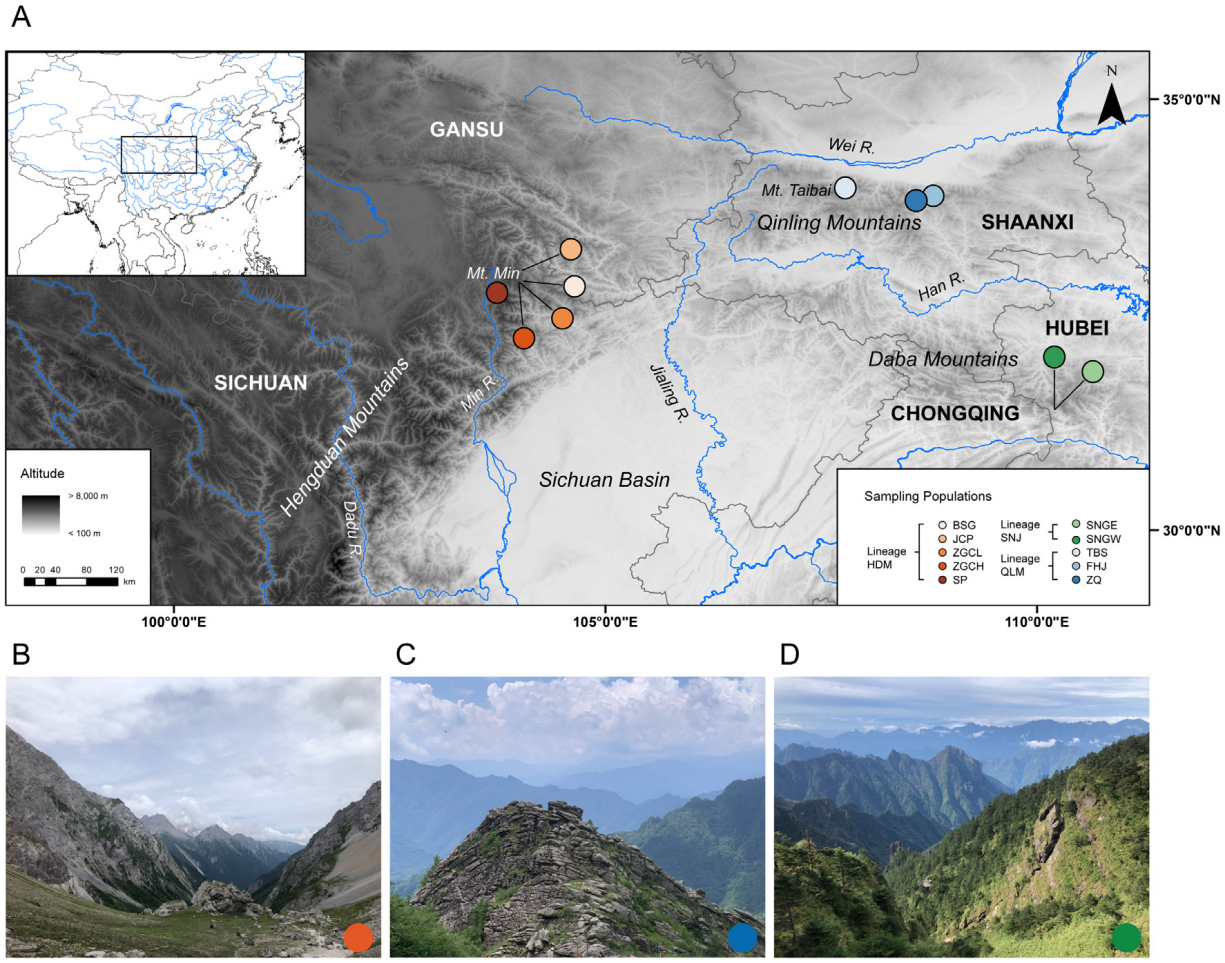
**(A)** Map of the study area showing the 10 sampling localities of *Chrysanthemum hypargyrum*. Colored circles represent different populations corresponding to each lineage. **(B)** Representative habitat of the HDM lineage. **(C)** Representative habitat of the QLM lineage. **(D)** Representative habitat of the SNJ lineage.

*C. hypargyrum* is a narrowly distributed species endemic to central China. A total of 106 individuals from 10 populations covering the entire range of this species were sampled in this study (**Table 1**; **Figure 1**). Additionally, three *Chrysanthemum* species with five sampled populations were used as outgroups for phylogenetic analyses, including one species from the *C. indicum* complex and two species from the *C. zawadskii* complex; our study species belonged to the *C. zawadskii* complex (**Table 1**). Voucher specimens were deposited at the Herbarium of Peking University (PEY). Young, fresh leaves from each sampled individual were collected in the field and preserved in silica gel until they were needed for DNA extraction. To determine the ploidy level across populations, some individuals from different populations and geographical localities were transplanted to our greenhouse for subsequent flow cytometric ploidy examination using a diploid individual of *C. hypargyrum* HDM (determined by chromosome counts) as a standard reference (refer to Li et al., 2013).

**Table 1:** Collection information and data for *Chrysanthemum hypargyrum* and outgroup taxa samples used in this study.

Taxon	Population	Location	Latitude (°N)	Longitude (°E)	Elevation (m)	Number of individuals	Voucher
*C. hypargyrum*							
Lineage HDM	BSG	Baishagou, Sichuan	32.88	104.05	2840	12	Rao-Hu202107a
	JCP	Jincaopo, Sichuan	33.00	104.03	2860	11	Rao-Hu202107b
	SP	Songpan County, Sichuan	32.75	103.75	3830	11	Rao-Meng201908a
	ZGCH	Zhugencha (high altitude), Sichuan	32.87	104.00	4055	8	Luo202210a
	ZGCL	Zhugencha (low altitude), Sichuan	32.87	104.00	3819	8	Luo202210a
Lineage SNJ	SNGE	Shennonggu (east), Hubei	31.44	110.28	2890	11	Hu202109a
	SNGW	Shennonggu (west), Hubei	31.44	110.27	2880	12	Hu202109b
Lineage QLM	TBS	Taibai mountains, Shaanxi	33.97	107.78	3700	11	Rao-Liu200507a
	FHJ	Fenghuangju, Shaanxi	33.87	108.80	2514	12	Hu-Rong202009a
	ZQ	Zhuque Forest Park, Shaanxi	33.82	108.60	2400	10	Zhang202009a
Outgroup taxa from *C. zawadskii* complex							
*C. oreastrum*	WTS	Wutai mountains, Shanxi	39.08	113.57	3048	1	Lu201806a
	XWT	Xiaowutai mountains, Hebei	39.94	115.04	2813	1	Lu201806a
	CBS	Changbai mountains, Jilin	42.06	128.26	1420	1	Shen-Zhang201609a
*C. chanetii*	XWT	Xiaowutai mountains, Hebei	40.04	115.05	1172	1	Lu201810a
Outgroup taxa from *C. indicum* complex							
*C. lavandulifolium*	PKU	Yanyuan, Beijing	39.99	116.30	92	1	Shen-Zhang201609a

### 2.2 Morphological characterization

To characterize the morphological divergence in *C. hypargyrum*, we characterized the leaf epidermis and performed comparative analyses of leaf shapes and ray floret color. For the leaf epidermal characterization, we collected the basal-most second and third leaves from two individuals from each geographic region within the range of this species. Microphotographs were captured from the abaxial and adaxial surfaces using a Quanta FEG 450 scanning electron microscope (FEI, Hillsboro, Oregon, USA). For the morphological characterization of leaf shapes in three geographic regions, we randomly selected two populations from each geographic region and five mature individuals from each population. The basal-most second and third leaves were collected from each individual. The leaves were flattened and dried in a specimen holder and then scanned using a Perfection V370 Photo scanner (Epson, Los Alamitos, California, USA) at a resolution of 600 dpi. Scanned images were pre-processed via Fiji. The contour, sinus, and tip of leaves were automatically extracted using Morpholeaf (Biot et al., 2016); Morpholeaf was also used to take measurements of leaf characteristics, including blade length, blade width, blade area, blade perimeter, and total number of teeth. The blade length/blade width ratio and the dissection index (blade perimeter/(4π × blade area)^1/2) were also calculated. These seven leaf traits were selected based on their diagnostic value in a previous study of *Chrysanthemum* species (Lu et al., 2022). Principal component analysis (PCA) of the seven leaf morphometric parameters was conducted using Origin version 9.9.0.225. All seven indices were plotted separately using the package ggplot2 (Wickham, 2016) in R version 4.2.2 (R Core Team, 2013). *P*-values were calculated using the Wilcoxon test for pairwise comparisons and Kruskal-Wallis test for multiple comparisons. Ray floret color was recorded from 15 capitula with three repeats using an AvaSpec-2048 spectrometer (Avantes, Beijing, China), and the chromaticity values were used to generate a scatter plot of the color distribution in SigmaPlot 15.0.

### 2.3 HyRAD library preparation and sequencing

We randomly selected one individual from the *C. hypargyrum* HDM lineage transplanted in our greenhouse as the source for the generation of homemade bait. Genomic DNA was extracted from silica-dried leaves using the Plant Genomic DNA Kit DP305 (TIANGEN Biotech, Beijing, China). Bait generation was carried out following a protocol modified from previously published protocols (Suchan et al., 2016; Lang et al., 2020). Briefly, we first digested DNA with the restriction enzymes *Eco*RI-HF R3101V and *Nsi*I-HF R3127V (New England Biolabs, Ipswich, MA, USA) and ligated custom adapters (APL6_EcoRI and APL5_NsiI) to the *Eco*RI/*Nsi*I-cut sticky ends of the digested fragments (Lang et al., 2020). For size selection, we performed agarose gel electrophoresis on the bait sample, and a 300 to 500-bp DNA smear was manually cut and purified using an AxyPrep DNA Gel Purification Kit AP-GX-250 (Axygen, Tewksbury, MA, USA). The size-selected bait sample was then amplified via PCR using custom primers (APL5 and APL6), which were designed for hybridization to the ligated adapters (Lang et al., 2020) and NEBNext Ultra II Q5 Master Mix M0544S (New England Biolabs, Ipswich, MA, USA). Adapters were removed from the PCR-amplified bait sample with *Eco*RI-HF and *Nsi*I-HF. Finally, we used the BioNick DNA Labeling System 18247015 (Invitrogen, Carlsbad, CA, USA) for bait biotinylation. The biotinylated bait was stored at -20°C until hybridization.

We extracted total genomic DNA from silica-dried leaves of 106 C*. hypargyrum* individuals and five outgroups using the Plant Genomic DNA Kit DP305 (TIANGEN Biotech, Beijing, China). The paired-end library was constructed using the Hieff NGS Onepot II DNA Library Prep Kit for Illumina 12204 (Yeasen Biotechnology, Shanghai, China) following the manufacturer’s protocol. For hybridization capture of the targeted DNA fragments from the genomic libraries, the TargetSeq One Kit (iGeneTech, Beijing, China) was used following the manufacturer’s protocol, except that the biotinylated probes in this procedure were replaced by our home-made baits. After library-bait incubation (60°C, 36 hr.), streptavidin bead capture, washing, and post-capture PCR amplification, the enriched libraries were sequenced (paired-end, 150 bp) on a NovaSeq 6000 (Illumina, San Diego, CA, USA) at GENEWIZ Inc. (Suzhou, Jiangsu, China) or ANOROAD Inc. (Beijing, China).

### 2.4 Read mapping and genotype calling

The quality of raw reads was assessed using FastQC version 0.11.9 (https://www.bioinformatics.babraham.ac.uk/projects/fastqc/). Illumina adaptors and low-quality bases were then removed using Trimmomatic version 0.39 (Bolger et al., 2014) with the following parameters: ILLUMINACLIP: TruSeq3-PE.fa:2:30:10:8:keepBothReads SLIDINGWINDOW:5:20 LEADING:3 TRAILING:3 MINLEN:36. Clean reads of each sample were mapped to the *Chrysanthemum lavandulifolium* reference genome (Wen et al., 2022) using BWA-MEM version 0.7.17 (Li and Durbin, 2009) with default settings. Binary alignment files were sorted with SAMtools version 1.15.1 (Li, 2011), and duplicated reads were marked and removed with Picard implemented in GATK version 4.2.6.1 (https://gatk.broadinstitute.org). Subsequently, single nucleotide polymorphism (SNP) calling was performed using GATK version 4.2.6.1. The retained SNP dataset was then filtered with BCFtools version 1.15.1 (Li, 2011) and VCFtools version 0.1.16 (Danecek et al., 2011) under the following criteria: (a) SNPs with quality score (Phred) < 30 were removed; (b) only biallelic sites were retained; (c) SNPs at or within 5 bp from any indels were removed; (d) no two SNPs were within 5 bp from one another; (e) SNPs with minor allele frequency < 0.05 were removed; (f) SNPs with depth of coverage (DP) lower than 3 were removed; and (g) SNPs with missing data > 50% were removed. Finally, a high-quality SNP dataset was retained for further analyses.

### 2.5 Phylogenetic analysis

Phylogenetic analyses were performed separately on the nuclear and plastid datasets. The filtered SNP dataset was used to reconstruct the nuclear phylogeny. The variant call format (VCF) file containing filtered SNPs was first converted to the PHYLIP format alignment file using vcf2phylip version 2.8 (Ortiz, 2019). In addition, we used IQ-TREE version 2.2.0.3 (Minh et al., 2020) to generate a maximum likelihood (ML) phylogenetic tree with the best model, TVM+F+ASC+R6, and 1,000 ultrafast bootstrap replicates.

To perform plastid phylogenetic analyses, a dataset containing plastid intergenic spacers (IGSs) was generated. First, we downloaded the complete plastid genome GenBank file of *C. zawadskii* from NCBI (accession number: NC_056150), after which all IGS sequences were extracted from the plastid genome. Next, the IGS dataset was introduced to the HybPiper version 2.1.1 pipeline (Johnson et al., 2016) as the target file, which served as “bait” to “capture” the off-target reads from hyRAD sequencing. A total of 118 IGSs from 111 individuals were assembled (**Supplementary Table S3**). Multiple sequence alignments were carried out using MAFFT version 7.508 (Katoh and Standley, 2013). A supermatrix with no more than 50% missing data was concatenated with aligned IGS sequences using the “pxcat” program implemented in Phyx (Brown et al., 2017a). The ML phylogenetic tree was reconstructed using IQ-TREE with 1,000 ultrafast bootstrap approximations and the best model, K3Pu+F+I+I+R3, was selected by ModelFinder.

### 2.6 Divergence time estimation

Given the large amount of nuclear data, we estimated divergence time using a reduced hyRAD dataset to accelerate calculations in BEAST version 1.10.4 (Suchard et al., 2018). This dataset consists of data from 25 samples, including 20 C*. hypargyrum* individuals (two randomly selected individuals from each population) and five outgroup samples. We performed divergence time estimation using the strict clock model with a clock rate of 1.66×10^−3^ substitutions/site/Myr and the speciation Yule model with a random starting tree (Lu et al., 2022). Substitution models for this dataset were assessed according to the Bayesian Information Criterion in ModelFinder, and the optimal model was GTR + I + G. Because of the lack of *Chrysanthemum* fossil evidence, we used secondary age estimates instead of fossil ages as calibration points. Two calibration points were selected according to Lu et al. (2022). First, we modeled a normal distribution prior for the crown age of *Chrysanthemum* with a mean age of 4.83 Mya and a standard deviation (SD) of 0.16 Mya. The other calibration point was the divergence time between the *C. oreastrum* WTS population and the XWT population, with a mean age of 1.41 Mya and a SD of 0.13 Mya. The MCMC chain was run for 1×10^8^ generations, and trees were sampled every 10,000 generations. Tracer version 1.7.2 (Rambaut et al., 2018) was used to check the convergence of each independent analysis (effective sample size values >200). Subsequently, a maximum clade credibility tree was constructed using TreeAnnotator version 1.10.4 (Suchard et al., 2018) with 10% of the trees discarded as burn-in. The final tree was visualized in FigTree version 1.4.3 (www.tree.bio.ed.ac.uk/software/figtree/).

### 2.7 Genetic diversity and population structure analysis

First, nucleotide diversity (*π*) and pairwise *F*_ST_ (between lineages and between populations within each lineage) was estimated using VCFtools. The observed heterozygosity (*H*_o_) and the expected heterozygosity (*H*_e_) for each population were calculated using Plink version 1.90 (Purcell et al., 2007). For population structure analysis, Plink was first used to apply linkage disequilibrium (LD) filtering on hyRAD data with the parameter –indep-pairwise 100 10 0.2, which generated an unlinked SNP subset for subsequent analyses. To evaluate the genetic relationships among samples, PCA was performed using the package SNPRelate (Zheng et al., 2012) in R version 4.2.2. Afterwards, we used fastSTRUCTURE (Raj et al., 2014) to visualize population structure with K-values ranging from 2 to 11. The Puechmaille method (Puechmaille, 2016) implemented in StructureSelector (Li and Liu, 2018) and the script “chooseK.py” implemented in fastSTRUCTURE were used to identify the K-value that was subsequently used to explain the structure in the data.

### 2.8 Demographic history inference

First, the multidimensional-folded site frequency spectrum (SFS) file was generated from hyRAD data using easySFS (https://github.com/isaacovercast/easySFS; Gutenkunst et al., 2009). A generation time of 3 years and a mutation rate of 4.98 × 10^−9^ per site per generation (Lu et al., 2022) were used for demographic history analyses. Subsequently, we used Stairway Plot 2 (Liu and Fu, 2020) to infer the evolutionary history of the three lineages of *C. hypargyrum*. Effective population sizes through time were estimated using 200 bootstrap replicates, and the precision of the estimations was evaluated using 95% confidence intervals. Tajima’s *D* for each lineage was estimated using VCFtools.

### 2.9 Analysis of the species distribution

The occurrence records of *C. hypargyrum* were collected from the Chinese Virtual Herbarium (https://www.cvh.ac.cn/), Chinese Field Herbarium (https://www.cfh.ac.cn/), Plant Photo Bank of China (http://ppbc.iplant.cn/), and our field investigations. Since there are a few known localities for this narrowly distributed species *C. hypargyrum*, we used all 10 known occurrence points for species distribution modeling (SDM) analysis, covering the entire distribution range of *C. hypargyrum* (**Figure 1**). The 19 bioclimatic variables were downloaded from the WorldClim database (http://www.worldclim.org) with a resolution of 30 arc-sec (Hijmans et al., 2005), and the relative contribution of each variable was estimated using jackknife tests. To avoid multicollinearity, pairwise Pearson correlation coefficients were calculated among the bioclimatic variables using ENMTools version 1.4.3 (Warren et al., 2010), and variables with relatively lower Pearson correlation coefficients (|*r*| < 0.8) and relatively higher contributions to the model were employed in the SDM analysis. Subsequently, we used the kuenm package (Cobos et al., 2019) in R version 3.6.3 (R Core Team, 2013) to conduct parameter tuning, and 255 candidate models were generated and tested by combining the 15 combinations of feature classes (linear, quadratic, product, and hinge) and the 17 regularization multipliers (0.1–1.0 in 0.1 increments, 2–6 in 1.0 increments, 8, and 10). Model performance was compared using three criteria: omission rate 5%, model complexity (AIC for small sample sizes), and significance (partial receiver operator characteristic). After determining the optimal model parameters, SDM was generated to simulate the distribution of *C. hypargyrum* for three periods, including the Last Glacial Maximum (LGM), mid-Holocene (MHO), and present day under the Community Climate System Model (CCSM) 4 (Braconnot et al., 2007) using Maxent version 3.3.4 (Phillips et al., 2006). The analysis was performed with 10 replicates and a 75%/25% training/testing partition of occurrence records (Lu et al., 2022; Qiao et al., 2018). Model performance was assessed using the area under the curve (AUC) of the receiver-operating characteristic (ROC) plot. To limit over-prediction, SDM results were pruned by a buffered minimum convex polygon (MCP) generated from the input occurrence data with a buffering distance of 5 decimal degrees using SDMtoolbox version 2.5 (Brown et al., 2017b). Maps of suitable areas were created using ArcGIS version 10.2.0.

## 3 Results

### 3.1 Morphological divergence between lineages

**Figure 2:**
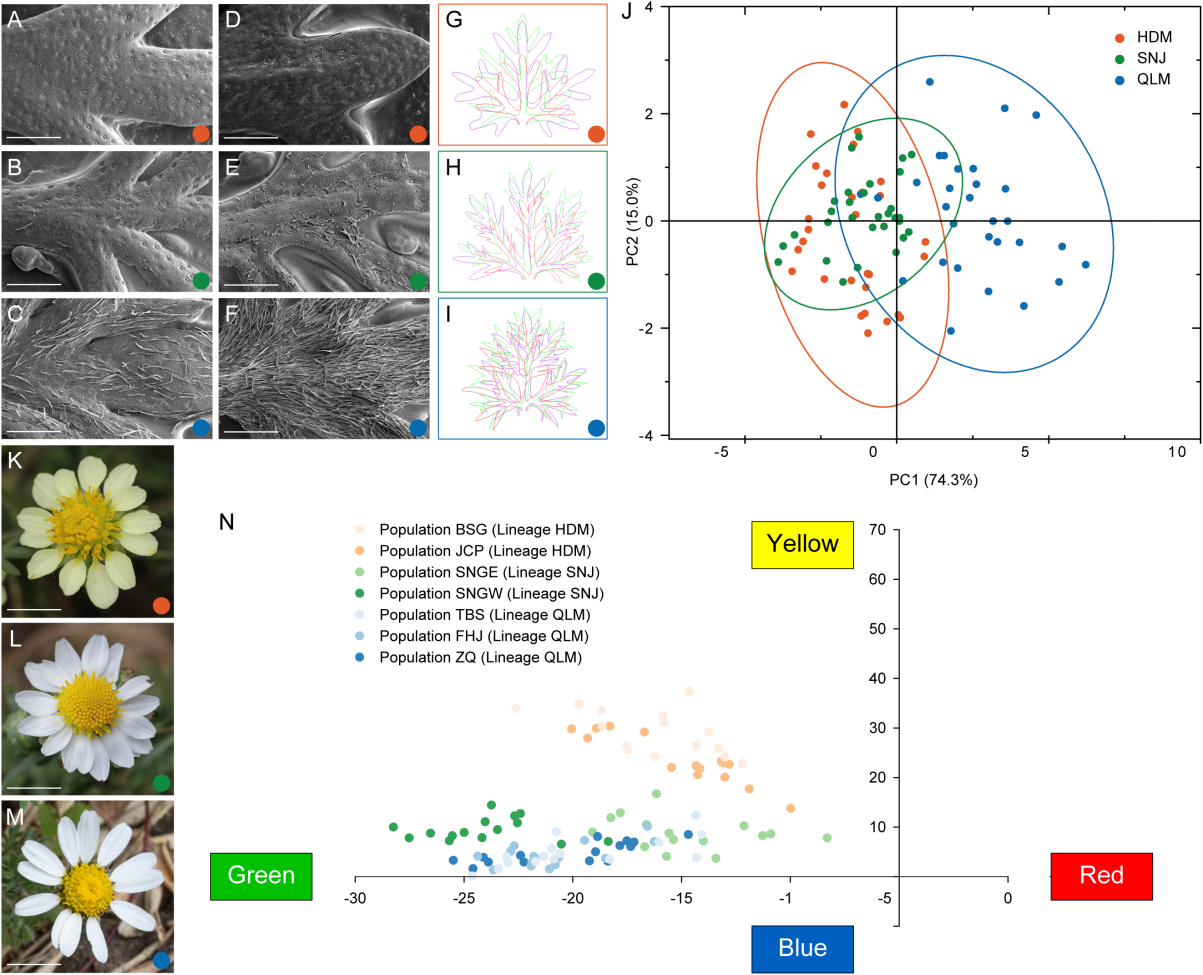
Morphological divergence between the three lineages of *Chrysanthemum hypargyrum*. **(A–F)** Scanning electron microscope (SEM) images of the adaxial **(A–C)** and abaxial **(D–F)** surface of leaves from the HDM **(A, D)**, SNJ **(B, E)**, and QLM **(C, F)** lineages. Scale bar, 1 mm. **(G**–**I)** Leaf contours of the HDM **(G)**, SNJ **(H)**, and QLM **(I)** lineages. The purple, green, and red outlines represent the range of variation in leaf contour within a lineage. **(J)** Biplot of principal component analysis (PCA) based on seven morphometric traits of the basal leaves of *Chrysanthemum hypargyrum*. Colored dots represent the three lineages. Ellipses represent 95% confidence intervals for each lineage of *Chrysanthemum hypargyrum*. **(K–M)** Floral morphology of HDM **(K)**, SNJ **(L)**, and QLM **(M)** showing ray floret color variation. Scale bar, 1 cm. **(N)** Quantitative analysis of ray floret color of *Chrysanthemum hypargyrum*. The vertical axis represents the yellowness value, and the horizontal axis represents the redness value.

Leaf epidermal characterization of *C. hypargyrum* revealed significant differences in epidermal trichome density between the three regions within its geographic distribution (**Figures 2A–F**). Trichomes were densest in the QLM, sparsest in the HDM, and intermediate in the SNJ. In addition, the trichomes on the abaxial surfaces were denser than those on the adaxial surfaces (**Figures 2A–F**). PCA of seven leaf morphometric indices revealed that QLM was separated from HDM and SNJ along PC1, but HDM and SNJ could not be effectively distinguished (**Figure 2J**). Specifically, QLM significantly differed from HDM and SNJ in seven leaf morphometric indices, but no significant difference between HDM and SNJ was observed in blade length, blade width, blade area, and blade length/blade width ratio (**Supplementary Figure S1**; **Supplementary Table S1**). For the quantitative analysis of ray floret color, the yellow ray florets of HDM were distinct from the white ones of SNJ and QLM, but little color divergence was observed between SNJ and QLM (**Figure 2N**).

### 3.2 HyRAD data and phylogenetic inference

An average of 15,953,186 (ranging from 8,276,797 to 51,541,819) clean reads per individual were retained for 111 hyRAD samples (**Supplementary Table S2**). Reads were mapped to the chromosome-level reference genome of *C. lavandulifolium*, and the mean mapping ratio was approximately 99.5% per sample (**Supplementary Table S2**). In the genotype calling dataset, the individual missing rate ranges from 0.071% to 0.24%, and the overall missing rate of the dataset is 0.14%. After filtering, 334,519 high-quality SNPs were obtained across all samples and retained for subsequent analyses.

**Figure 3:**
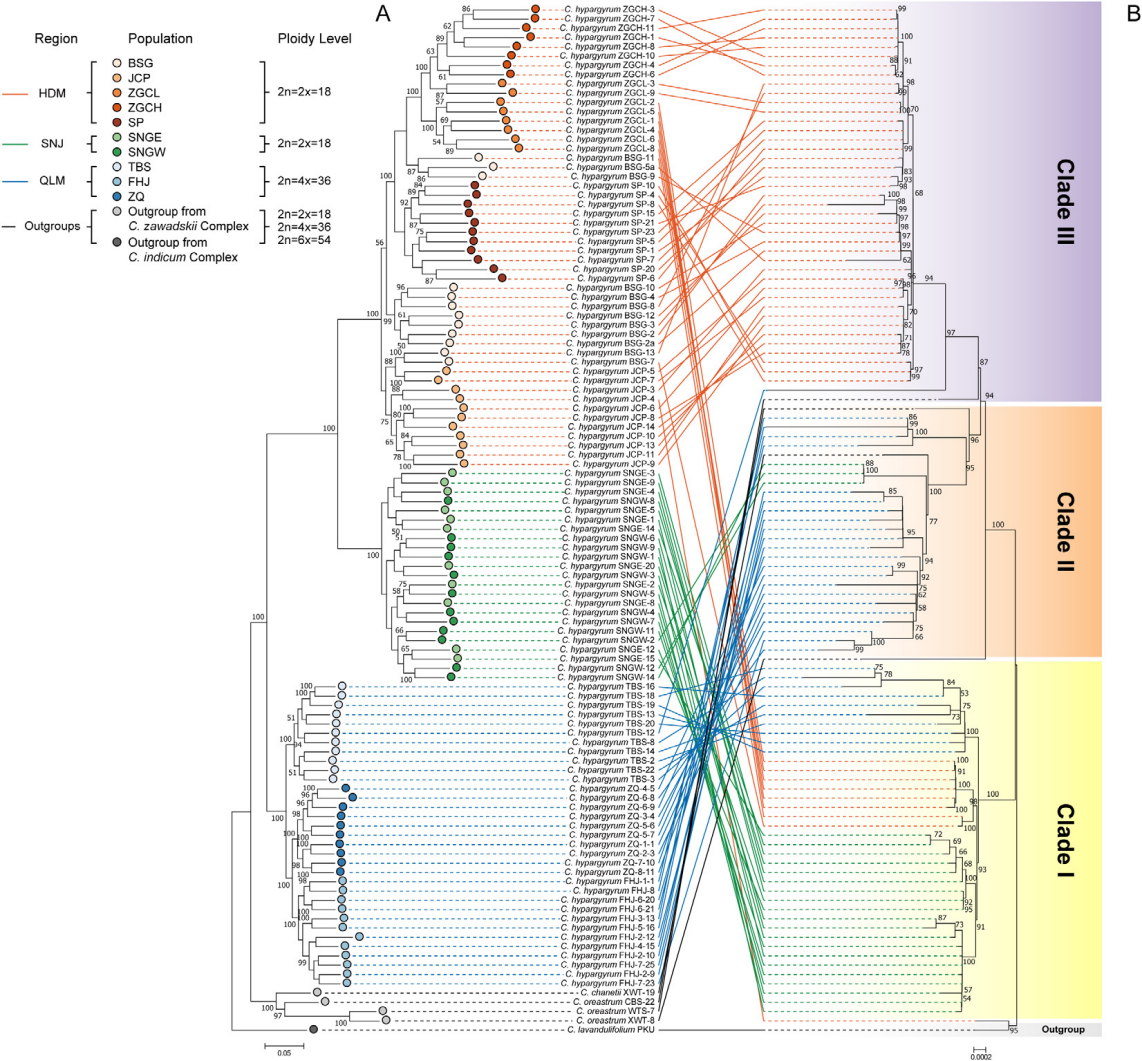
Phylogenetic relationships of *Chrysanthemum hypargyrum*, with five sampled *Chrysanthemum* outgroups. **(A)** Maximum likelihood tree based on nuclear single nucleotide polymorphisms (SNPs). **(B)** Concatenated maximum likelihood tree based on plastid intergenic spacers (IGSs). The numbers at nodes refer to bootstrap support values (support values below 50 not shown); colored circles and solid lines indicate the population and group assignments of the corresponding lineages, while gradient rectangles indicate the clade assignments of the plastid phylogeny.

The intraspecific phylogeny of *C. hypargyrum* was reconstructed with filtered SNPs obtained from the hyRAD dataset. For the hyRAD nuclear tree, all samples from the same geographic distribution range comprised a single clade (**Figure 3A**); *C. hypargyrum* thus comprised three clades corresponding to the three geographic regions (HDM, SNJ, and QLM). The QLM was the earliest diverging clade, and it was sister to the HDM-SNJ clade. HDM and SNJ were sister groups. QLM consists of three populations, TBS, FHJ, and ZQ, and the TBS population was the earliest diverging clade. SNJ comprised two populations, SNGW and SNGE, and they were not fully diverged (**Figure 3A**). HDM comprised five populations, and the JCP population was the earliest diverging clade. The ZGCH, ZGCL, and SP populations were clustered, and ZGCH and ZGCL were sister lineages (**Figure 3A**). The BSG population, a special population of the HDM, appeared at several positions on the phylogenetic tree (**Figure 3A**), suggesting that this could be an admixed population.

The plastid phylogenetic tree derived from IGS sequences can also be divided into three clades (**Figure 3B**), but there was incongruence between the plastid and nuclear hyRAD phylogenetic trees (**Figures 3A, B**). Clade I of the plastid tree comprised samples from all three geographic regions, which included a majority of SNJ individuals; individuals from the TBS population of the QLM; as well as individuals from the BSG and ZGCL populations of the HDM (**Figure 3B**). Clade II mainly comprised individuals from the QLM, as well as three individuals from the SNJ and two outgroup individuals from the *C. oreastrum* population CBS and the *C. chanetii* population XWT of the *C. zawadskii* species complex (**Figure 3B**). Clade III mostly comprised individuals from the HDM, as well as one individual from the QLM (TBS population) and one individual from the *C. oreastrum* WTS population (**Figure 3B**).

### 3.3 Diversification of *C. hypargyrum*

**Figure 4:**
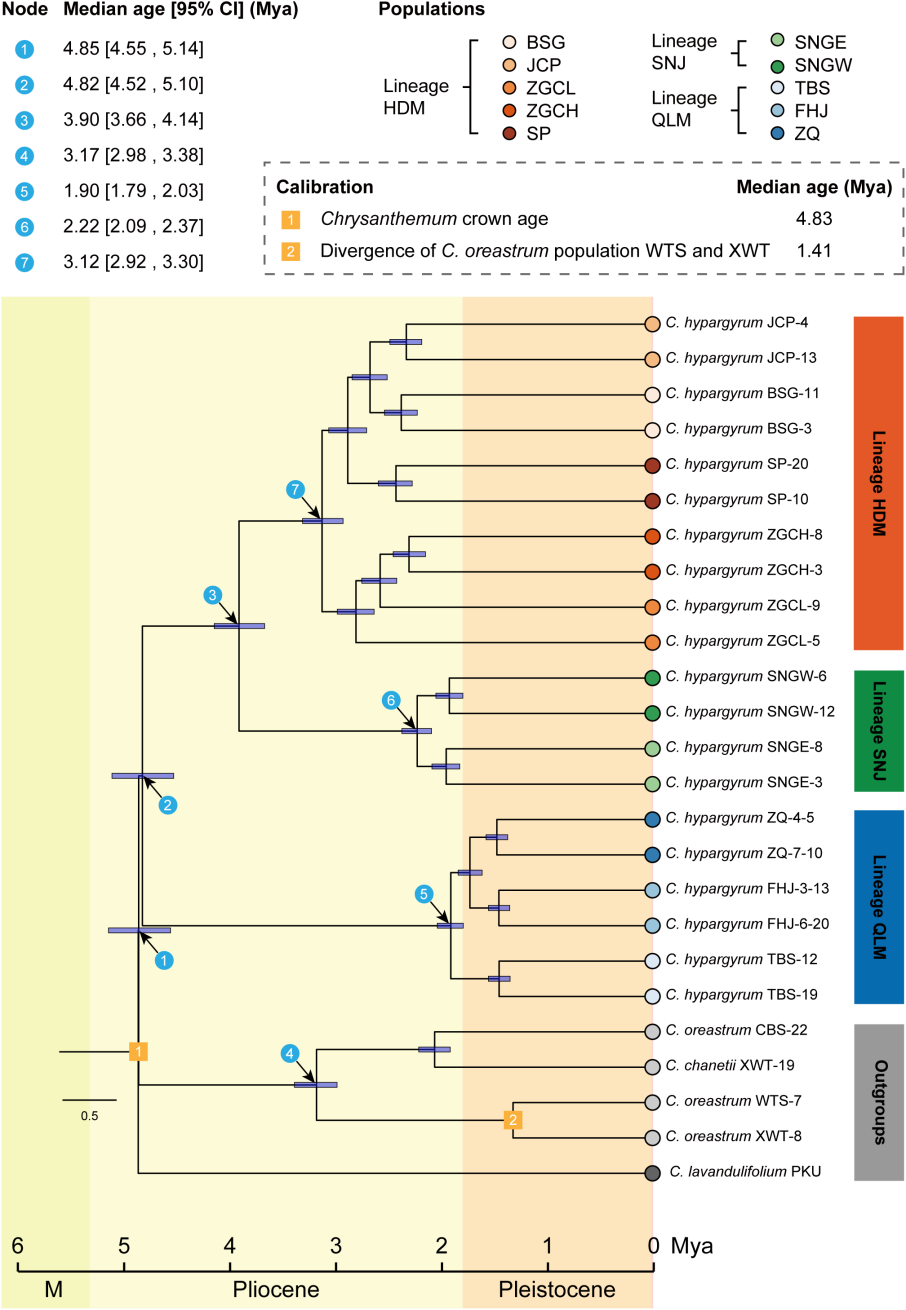
Divergence time estimations for major clades (blue circled numbers) of *Chrysanthemum hypargyrum* and the outgroup taxa.

The divergence time estimation showed that the *C. zawadskii* complex immediately diversified after the divergence between the *C. zawadskii* complex and the *C. indicum* complex of the genus *Chrysanthemum* at approximately 4.85 Mya (95% HPD, 4.55–5.14 Mya) (Node 1, **Figure 4**). The QLM populations of *C. hypargyrum*, which is the earliest diverging species of the *C. zawadskii* complex (Lu et al., 2022), split from the others right after the divergence of this species complex in the early Pliocene ca. 4.82 Mya (Node 2, **Figure 4**), and the HDM and SNJ populations diverged at 3.90 Mya (Node 3, **Figure 4**). The QLM, SNJ, and HDM clades all emerged in the Pliocene at 1.90 Mya (Node 5), 2.22 Mya (Node 6), and 3.12 Mya (Node 7), respectively (**Figure 4**).

### 3.4 Genetic diversity and population genetic structure

For five populations of the lineage HDM, the nucleotide diversity (*π*) ranged from 6.43×10^-6^ to 6.65×10^-6^, the observed heterozygosity (*H*_o_) ranged from 0.208 to 0.276, and the expected heterozygosity (*H*_e_) ranged from 0.162 to 0.212 (**Table 2**). For two populations of the lineage SNJ, the nucleotide diversity was 6.06×10^-6^ and 5.99×10^-6^, the observed heterozygosity was 0.257 and 0.268, and the expected heterozygosity was 0.194 and 0.202, respectively (**Table 2**). For three populations of the lineage QLM, the nucleotide diversity ranged from 4.68×10^-6^ to 4.96×10^-6^, the observed heterozygosity ranged from 0.233 to 0.244, and the expected heterozygosity ranged from 0.172 to 0.180 (**Table 2**). Lineage HDM had the highest level of genetic diversity, while lineage QLM had the lowest and lineage SNJ in between.

**Table 2:** Genetic diversity statistics for each population of *Chrysanthemum hypargyrum*.

Lineage	Population	Sample size	*π*	*H*_o_	*H*_e_
HDM	BSG	12	6.46E-06	0.276	0.212
	JCP	11	6.43E-06	0.274	0.209
	SP	11	6.55E-06	0.255	0.198
	ZGCH	8	6.55E-06	0.208	0.162
	ZGCL	8	6.65E-06	0.216	0.168
SNJ	SNGE	11	6.06E-06	0.257	0.194
	SNGW	12	5.99E-06	0.268	0.202
QLM	TBS	11	4.68E-06	0.244	0.180
	FHJ	12	4.96E-06	0.233	0.174
	ZQ	10	4.91E-06	0.234	0.172

*π*, nucleotide diversity; *H*_o_, observed heterozygosity; *H*_e_, expected heterozygosity.

**Figure 5:**
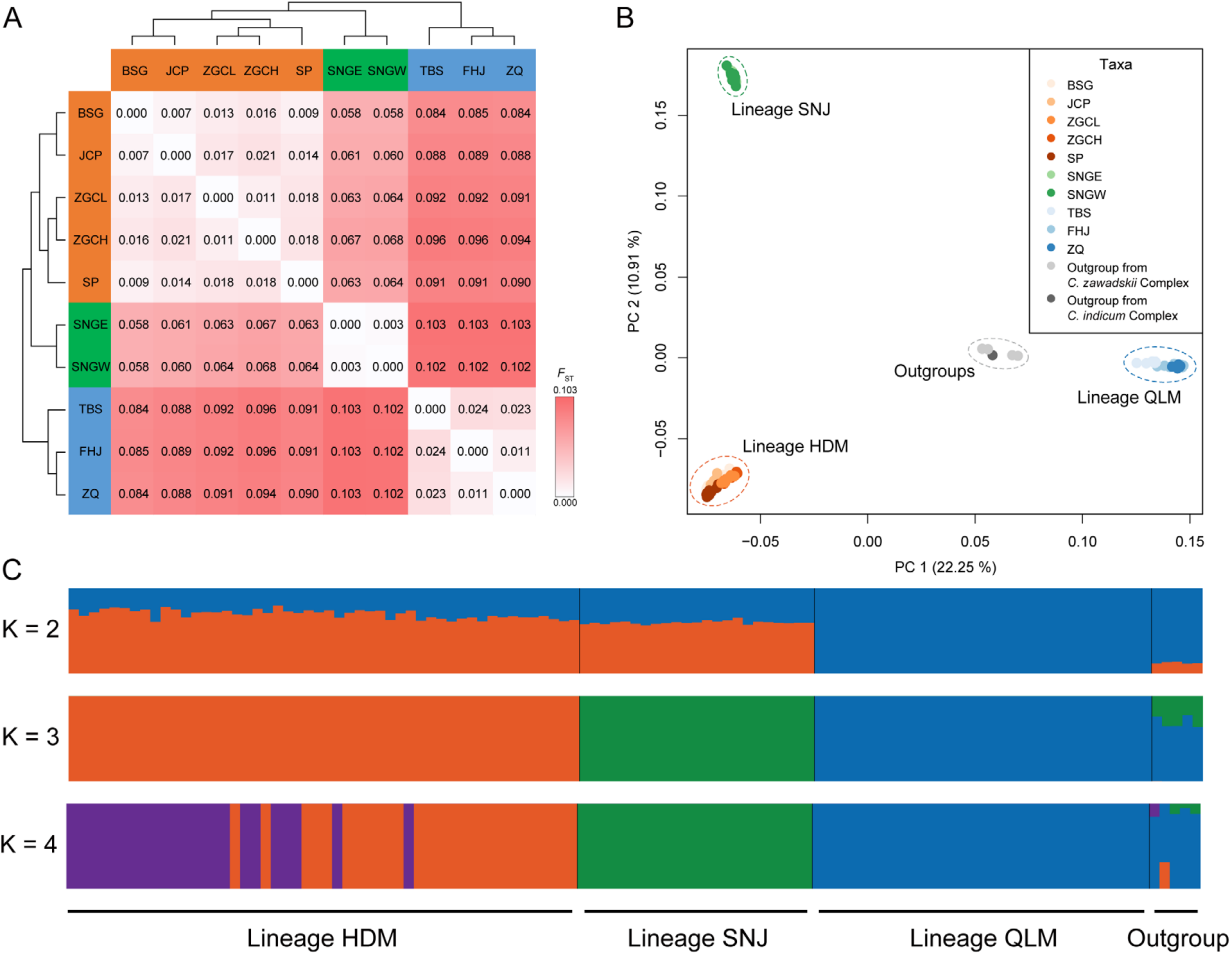
Population structure of *Chrysanthemum hypargyrum*, with five sampled *Chrysanthemum* outgroups. **(A)** Pairwise *F*_ST_ between *Chrysanthemum hypargyrum* populations. **(B)** Principal component analysis (PCA) biplot with the first two principal components based on genome-wide SNP data. **(C)** Bayesian model-based clustering analysis, when K = 2, 3 (optimal K-value), and 4. Each vertical bar represents one individual, and the x-axis shows the three lineages of *Chrysanthemum hypargyrum* and the outgroups. Each color represents one putative ancestral background, and the y-axis represents the probability of assignment to that cluster.

Pairwise *F*_ST_ analysis of populations showed that the genetic divergence between geographic regions was higher than that between populations within the same region (**Figure 5A**). Moreover, the mean pairwise *F*_ST_ between the three regions was 0.063 between HDM and SNJ, 0.090 between HDM and QLM, and 0.102 between SNJ and QLM, respectively, suggesting that SNJ populations were genetically more similar to HDM populations than QLM populations (**Figure 5A**).

PCA divided most samples into three groups: the HDM-SNJ, the QLM, and the outgroups along PC1 (variance explained = 22.25%). Further clustering separated HDM and SNJ along PC2 (variance explained = 10.91%) (**Figure 5B**).

The results of population genetic structure analysis of this species using fastSTRUCTURE were similar to the PCA results. Samples from the QLM were first separated from those of the HDM and SNJ regions when K = 2 (**Figure 5C**). When K = 3, all sampled individuals were assigned to three genetic clusters corresponding to the HDM, SNJ, and QLM lineages, with outgroups forming a distinct fourth cluster; no signs of admixture were detected (**Figure 5C**). When K = 4, genetic heterogeneity was observed among individuals in the HDM; in the SNJ and QLM, sampled individuals were genetically homogeneous (**Figure 5C**). In summary, both PCA and fastSTRUCTURE analyses strongly indicate that K = 3 is the optimal K-value, and *C. hypargyrum* has diverged into three lineages corresponding to the HDM, SNJ, and QLM.

### 3.5 Demographic history inference

**Figure 6:**
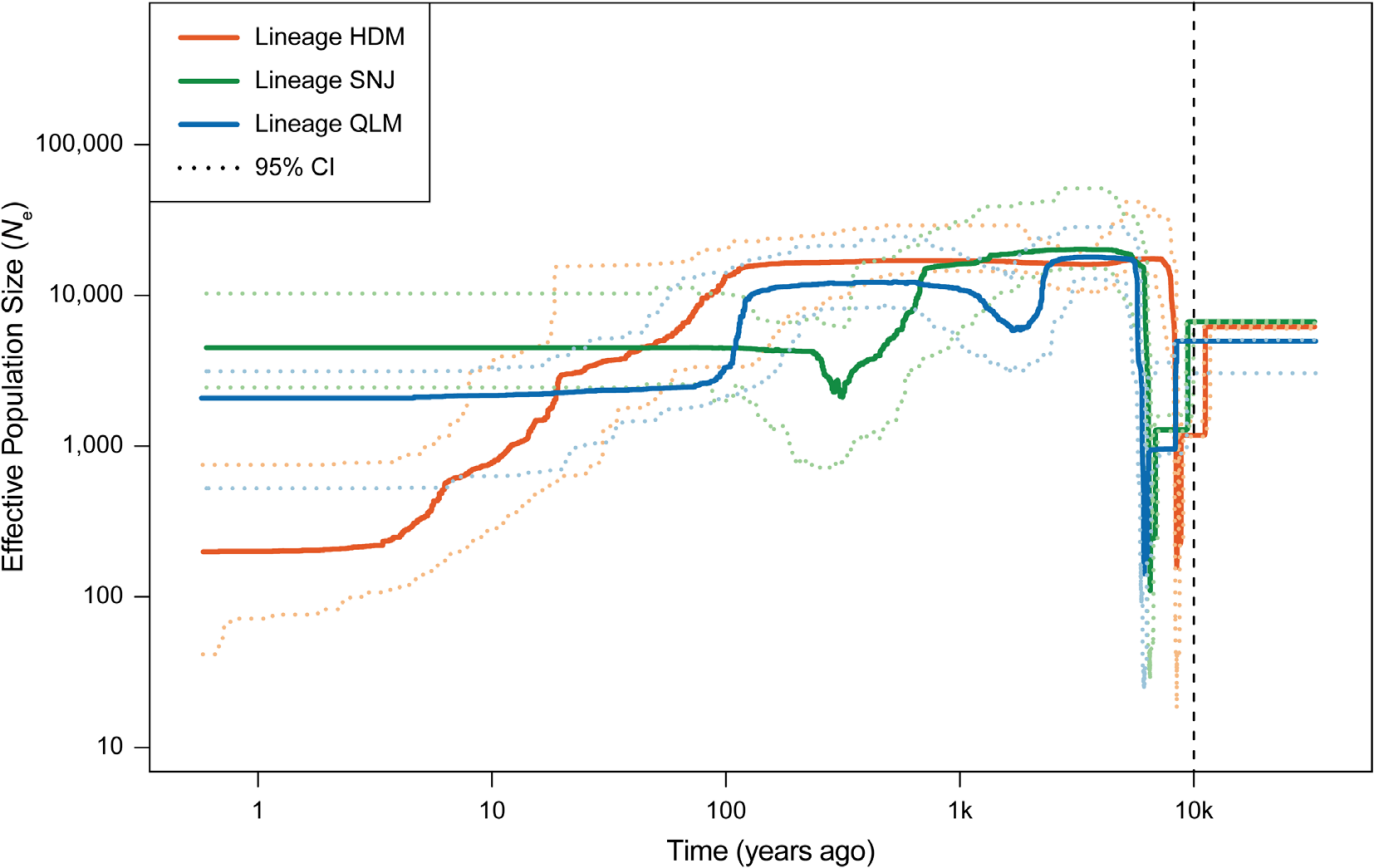
Stairway plot showing the demographic history of the three lineages of *Chrysanthemum hypargyrum*. Solid curves show the median of effective population size (*N*_e_) over time, and the dashed curves show the upper and lower 95% confidence intervals (CI) of the effective population size of the corresponding lineage. The vertical dashed line indicates the beginning of the Holocene (~10,000 years ago).

A stairway plot revealed that the three lineages of *C. hypargyrum* shared a highly similar pattern of historical oscillations in the effective population size (*N*_e_) over time. These three lineages underwent a dramatic decline in *N*_e_ but recovered shortly after this decline (**Figure 6**). Both the decline and the recovery happened around 10,000 years ago after the LGM or at the beginning of the Holocene. Aside from this drastic population shrinkage and expansion, within the last thousand years, the *N*_e_ of the HDM decreased slowly, and the population size of the SNJ and QLM was relatively stable despite some fluctuations (**Figure 6**). Thus, the mean Tajima’s *D* values of the three lineages were all below zero (**Table 3**), suggesting that population expansion occurred after a recent bottleneck.

**Table 3:** Mean Tajima’s *D* of the three lineages of *Chrysanthemum hypargyrum*.

Lineage	HDM	SNJ	QLM
Mean Tajima's *D*	-0.9978	-0.6914	-0.9467

### 3.6 Analysis of the distribution and dispersal of species

**Figure 7:**
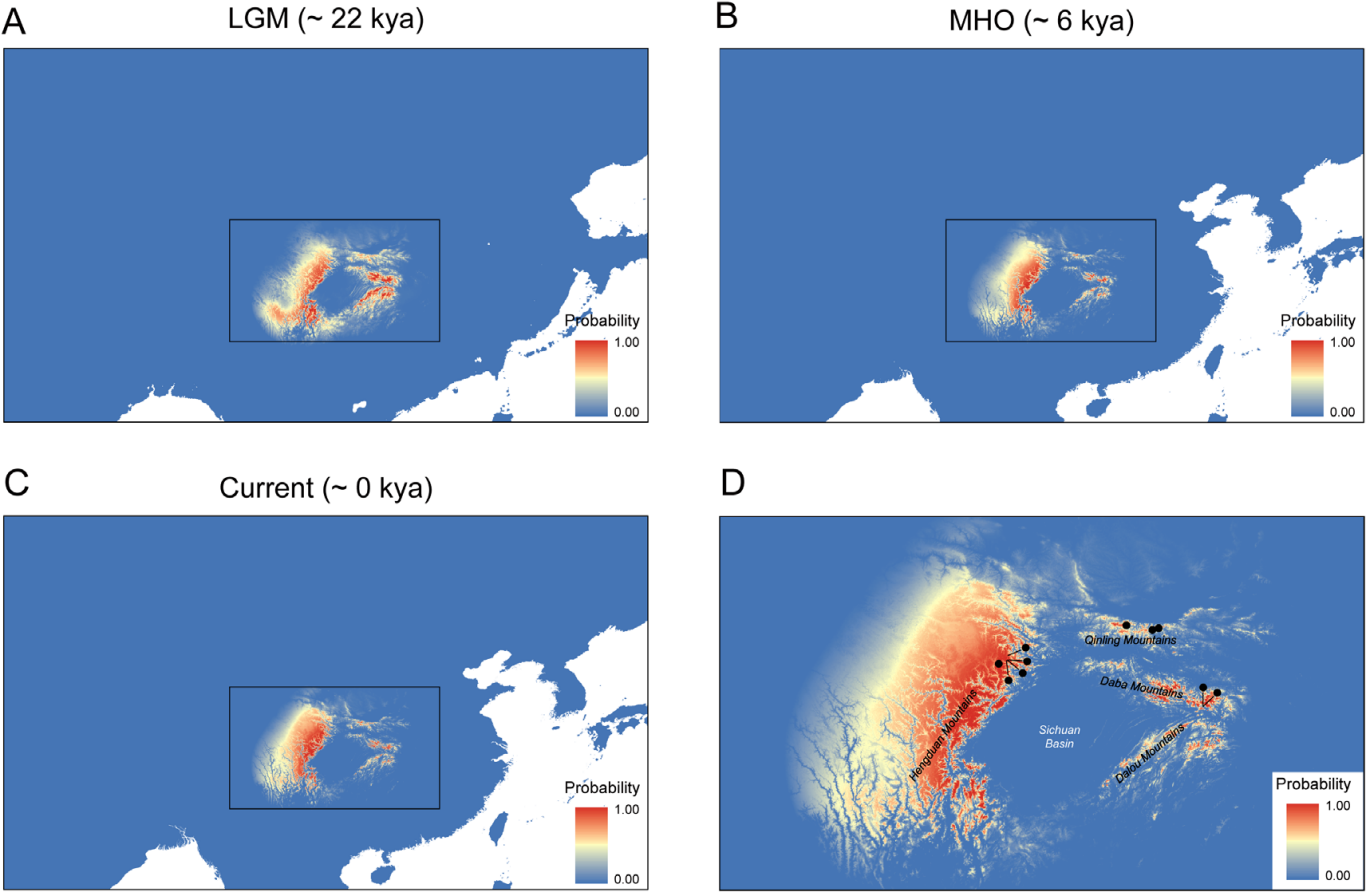
*Chrysanthemum hypargyrum* distribution models for current and different geological periods. **(A)** Distribution model for the Last Glacial Maximum (LGM) **(B)** Distribution model for the mid-Holocene (MHO). **(C)** Distribution model for the current period. **(D)** Magnified view of the boxed regions from **(A–C)**, showing major topographic features and sampling locations indicated by black dots. Red represents a high probability of distribution, while blue indicates the opposite.

After filtering, five environmental variables including BIO4 (temperature seasonality), BIO1 (annual mean temperature), BIO15 (precipitation seasonality), BIO12 (annual precipitation), and BIO19 (precipitation of coldest quarter) were considered as the best-performing predictors, and they were further used in our SDM analysis. The percent contribution and permutation importance of these best-performing predictors were listed in **Supplementary Table S4**. Among the environmental variables, BIO4 (temperature seasonality) contributed most significantly to the model (47% percent contribution), reflecting the critical role of temperature variability in restricting *C. hypargyrum* to high-elevation habitats. BIO1 (annual mean temperature) and BIO15 (precipitation seasonality) also showed substantial contributions (33.6% and 10.9% percent contribution, respectively), suggesting the combined effects of thermal and hydrological factors in maintaining sky island distribution. The final SDM result had an AUC > 0.9, indicating a high predictive ability and low degree of overfitting. Current climatically suitable areas predicted by the SDM yielded distribution patterns that were generally consistent with the actual current distribution of *C. hypargyrum*, which occupied the HDM, QLM, and the SNJ and its surrounding mountains, namely the Daba Mountains and the Wushan Mountains (**Figures 7C, D**). During the MHO, the distribution of this species was largely consistent with its present distribution, but it was a little smaller (**Figure 7B**). However, during the LGM, the distribution of *C. hypargyrum* expanded southward and extended to the Dalou Mountains, the southern edge of the Sichuan Basin (**Figures 7A, D**). Furthermore, *C. hypargyrum* can be inferred to be a cold-adapted species according to the SDM results during the LGM (**Figure 7A**).

## 4 Discussion

### 4.1 HyRAD, a powerful tool for the study of phylogeography, speciation, and population demography

Reduced representation sequencing (RRS) offers a cost-effective approach for studies of the phylogeny, phylogeography, and population demography of non-model organisms, especially species with large genomes (Hess et al., 2020; Christiansen et al., 2021; Ulaszewski et al., 2021). RRS includes a variety of approaches, and the most popular ones are GBS, RAD, and ddRAD. Despite their many advantages, RRS approaches are susceptible to missing data due to random mutations at restriction enzyme cutting sites among samples, a phenomenon known as allele dropout (Mastretta-Yanes et al., 2015; Andrews et al., 2016). Moreover, the flanking sequences near the enzyme cutting sites cannot be obtained through RRS approaches, which limits their sequence acquisition ability.

Target DNA enrichment by hybridization capture can obtain highly similar sequences near the target DNA sites and their flanking sequences. In this study, we performed hyRAD, a technology combining DNA hybridization and traditional RRS, which could enrich RAD sequences. HyRAD has the advantage of the high genome-level coverage of RRS (Suchan et al., 2016; Linck et al., 2017; Lang et al., 2020) and overcomes the shortcomings associated with allele dropout; it can also finally acquire flanking sequences near the enzyme cutting sites (Andrews et al., 2016; Suchan et al., 2016). HyRAD not only recovered 24% more SNPs than traditional RAD methods (Lang et al., 2020), but also exhibited a remarkably low missing rate (individual missing rate: 0.071–0.24%; overall missing rate: 0.14%). Therefore, our results show that hyRAD is a successful and powerful approach for studying the demographic history and speciation of non-model species by obtaining vast amounts of homologous genomic-scale data compared with traditional RRS methods.

### 4.2 Morphological divergence and taxonomic delineation of *C. hypargyrum*

Morphological divergence is one of the key criteria for taxonomic delineation (De Queiroz, 2007; Sites and Marshall, 2004). Morphology is determined by both genes and the environment (West-Eberhard, 2003). Firstly, genetic divergence is one of the prerequisites for morphological divergence, hence the degree of morphological divergence between lineages can reflect, to some extent, their genetic divergence (Slatkin, 1987). Conversely, morphological divergence may lead to changes in the adaptability of different lineages to various environments. To accurately determine the relationship between morphological and genetic divergence, it is necessary to obtain both morphological and genetic data for the same taxon (García et al., 2008; Garnier et al., 2005; Ortego et al., 2012). This study has obtained both morphological and genetic data for *C. hypargyrum*, aiming to understand the degree of morphological and genetic divergence among its three lineages, as well as the inter-lineage relationship. The morphological data we obtained consists of multiple dimensions, including morphometric analyses of leaf shapes, leaf epidermal characterization, and quantitative analysis of ray floret color, while the genetic data mainly includes nuclear hyRAD data and plastid IGS data. Overall, the three lineages of *C. hypargyrum* have undergone a certain degree of morphological differentiation, meaning that it is relatively easy to distinguish the three lineages morphologically. Considering that three lineages of *C. hypargyrum* were ever considered as different species in previous taxonomic works (Chang, 1934; Ling and Shih, 1980; Shi et al., 2011), we can conclude that, at least from the perspective of morphological divergence, and a further taxonomic revision of this species is necessary.

### 4.3 Origin and evolutionary history of *C. hypargyrum*

According to the hyRAD phylogenetic tree, which contained three lineages of *C. hypargyrum*, the QLM lineage diverged first, and this was followed by the divergence between the HDM and SNJ lineages. This supported the conclusion made by Lu et al. (2022) that the Qinling–Daba Mountains and its adjacent area could be the origin of the *C. zawadskii* complex. Given that *C. hypargyrum* is the most basal lineage of the *C. zawadskii* complex, it can be inferred by our present findings that *C. hypargyrum* possibly originated from the Qinling–Daba Mountains and later migrated to the HDM and SNJ. According to the plastid IGS phylogeny of *C. hypargyrum*, the earliest diverged clade I contains most of the individuals from lineage SNJ, suggesting a more ancestral origin of the lineage SNJ. Additionally, based on the phylogenetic results from different datasets (**Figure 3**), either the QLM lineage or the SNJ lineage was more closely related to the more distantly-related lineage of *C. hypargyrum* (Omland et al., 2008; McDaniel, 2021; Walker et al., 2024). Based on the ploidy pattern in the three lineages of *C. hypargyrum*, the tetraploidy of the QLM lineage, and the diploidy of the HDM and SNJ lineages, the most recent common ancestor of these lineages was diploid. The SNJ is on the east end of the Daba Mountains, and the QLM and the Daba Mountains are closely related (often collectively referred to as the Qinling–Daba Mountains). Therefore, the Daba Mountains serve as the hub connecting the QLM and the SNJ, which is the most likely ancestral origin of the diploid ancestor of *C. hypargyrum*. The Daba Mountains’ role as a biogeographic hub is further supported by ancestral area reconstruction in Lu et al. (2022), which identified this region as the ancestral origin of the *C. zawadskii* complex. Additionally, the divergence time of *C. hypargyrum* lineages aligns with the uplift of the Qinling–Daba Mountains in late Miocene to Pleistocene (Shi et al., 2020), suggesting that tectonic activity created both physical and climatic barriers that facilitated allopatric divergence.

Mountain-building processes and other tectonic movements are well-known speciation drivers (López-Pujol et al., 2011). According to the divergence time estimation (**Figure 4**), the formation of *C. hypargyrum* as well as its three major lineages all occurred in the Pliocene, and mountain uplifts frequently occurred in this period (**Figure 4**). For example, the uplift of the Qinling–Daba Mountains from the late Miocene to the Pleistocene (Liu et al., 2013; Meng, 2017; Shi et al., 2020) may have facilitated the allopatric divergence of the QLM lineage with the other two, which happened approximately 4.82 million years ago (**Figure 4**). Additionally, the bifurcation of the HDM and SNJ lineages was estimated to have occurred around 3.90 million years ago (**Figure 4**), and rapid mountain uplifts occurred in the HDM and SNJ (3.4–3.0 Mya), which established geographical barriers to gene flow and subsequent genetic divergence between regions (Xing and Ree, 2017; Ding et al., 2020; Shi et al., 2020).

### 4.4 Cytonuclear incongruence in the phylogeny of *C. hypargyrum*

Discordance between nuclear and plastid phylogenies, a phenomenon known as cytonuclear incongruence, is frequently observed in molecular phylogenetics and poses a major challenge for understanding the evolutionary history of plants (Gitzendanner et al., 2018; Lee-Yaw et al., 2019; Lu et al., 2022). In this study, cytonuclear incongruence was primarily characterized by the fact that samples from three geographical regions can be clustered together in the nuclear phylogenetic tree according to their respective regions; however, in the plastid phylogenetic tree, a clade can have samples from different geographical regions (**Figure 3B**). This phenomenon can be attributed to ancestral polymorphism retention, localized introgression events, or both. The cytonuclear incongruence in this study indicates that limited historical gene flow could occur because of shared haplotypes across lineages, particularly between the SNJ, HDM, and QLM lineages of Clade I. In addition, lineage-specific discordance can be inferred from the absence of HDM samples in plastid Clade II and SNJ samples in Clade III (**Figure 3B**).

### 4.5 The role of “sky islands” in the evolution of *C. hypargyrum*

As mentioned above, phylogenetic analysis and ancestral area reconstruction in Lu et al. (2022) revealed a “stepping-stone-like” migration model for the sky island distribution of *C. hypargyrum*. A similar model is also supported by many animal taxa, such as spiders, salamanders, shrews, and moles (Chen et al., 2015; He et al., 2019; Hedin et al., 2015; Shepard and Burbrink, 2008). However, relevant studies based on plant species are still scarce. Our findings show that *C. hypargyrum* provides a good example of a plant species with a sky island distribution.

Quaternary climatic oscillations have greatly influenced patterns of biodiversity and the distributions of plants in the Northern Hemisphere, especially in mountainous areas (Ren et al., 2017; Đurović et al., 2021; Ortego and Knowles, 2022). Many organisms in this region have undergone major habitat shifts during glacial–interglacial cycles. These shifts caused the separation, migration, and extinction of populations, which accelerated the rate of evolution. Alpine species, such as *C. hypargyrum*, experienced dynamic changes in suitable habitats during these cycles. SDM results indicate that the predicted distribution ranges of all three lineages of *C. hypargyrum* expanded during the LGM and contracted during the MHO, and they are currently isolated in three discontinuous mountainous areas: the HDM, QLM, and SNJ (**Figure 7**). For some alpine species, the displacement of sky island habitats to lower elevations during past glacial periods may have caused range overlap and secondary contact between formerly separated populations, which altered the isolated distribution of sky island species (Hewitt, 1996; DeChaine and Martin, 2004; Galbreath et al., 2009; McCormack et al., 2009). However, regardless of the observed distributional changes during the glacial–interglacial cycles, three lineages of *C. hypargyrum* remained isolated in a sky island pattern. This suggests that the climate oscillations did not disrupt but rather maintained the sky island distribution of *C. hypargyrum*.

Geographic isolation is usually regarded as a fundamental factor underlying the genetic diversification of a species (Slatkin, 1993; Bradburd et al., 2013; Wang and Bradburd, 2014). The distribution expansion of *C. hypargyrum* during cold period, as well as its cold-adapted nature facilitated the geographic isolation and the formation of three lineages of this species. The sky island distribution pattern further promoted the diversification and phylogeographic structure of *C. hypargyrum*.

## Data Availability

The data presented in the study are deposited in the Genome Sequence Archive (GSA) of China National Center for Bioinformation (CNCB), BioProject accession number PRJCA037776.
