# Supplementary material for: Hybridization ddRAD‐sequencing and phenotypic analysis clarify the phylogeographic structure and evolution of an alpine Chrysanthemum species with a sky island distribution
Source: Front Plant Sci. 2025 Apr 7;16:1563127. doi: 10.3389/fpls.2025.1563127 (PMC12009766; doi:10.3389/fpls.2025.1563127)
Supplement: Supplementary file 1 [file DataSheet1.docx]

Supplementary Material

# Supplementary Figures
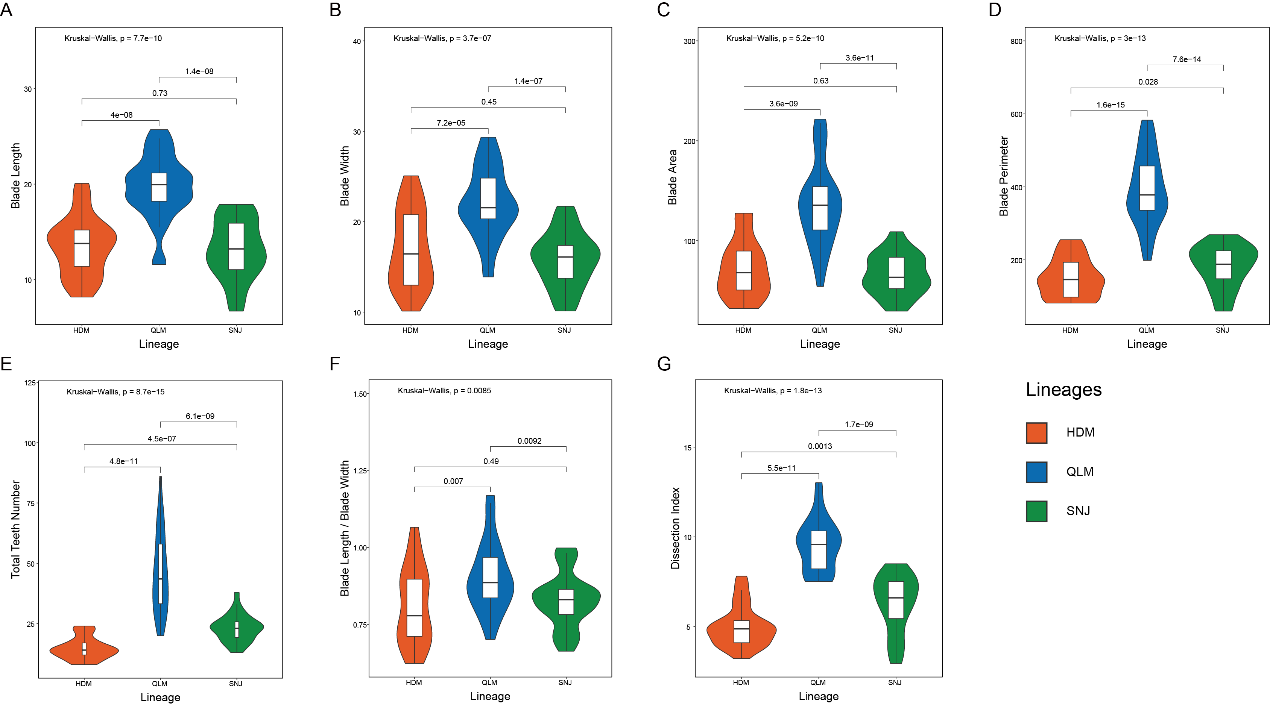


**Supplementary Figure 1.** Violin plots showing the distribution of the seven leaf morphometric statistics among the three lineages of *Chrysanthemum hypargyrum*. Box plots in each violin plot depict the median, 1st, and 3rd quartiles for each distribution. P values were calculated using Wilcoxon test for pairwise comparison test and Kruskal-Wallis test for multiple comparison test.


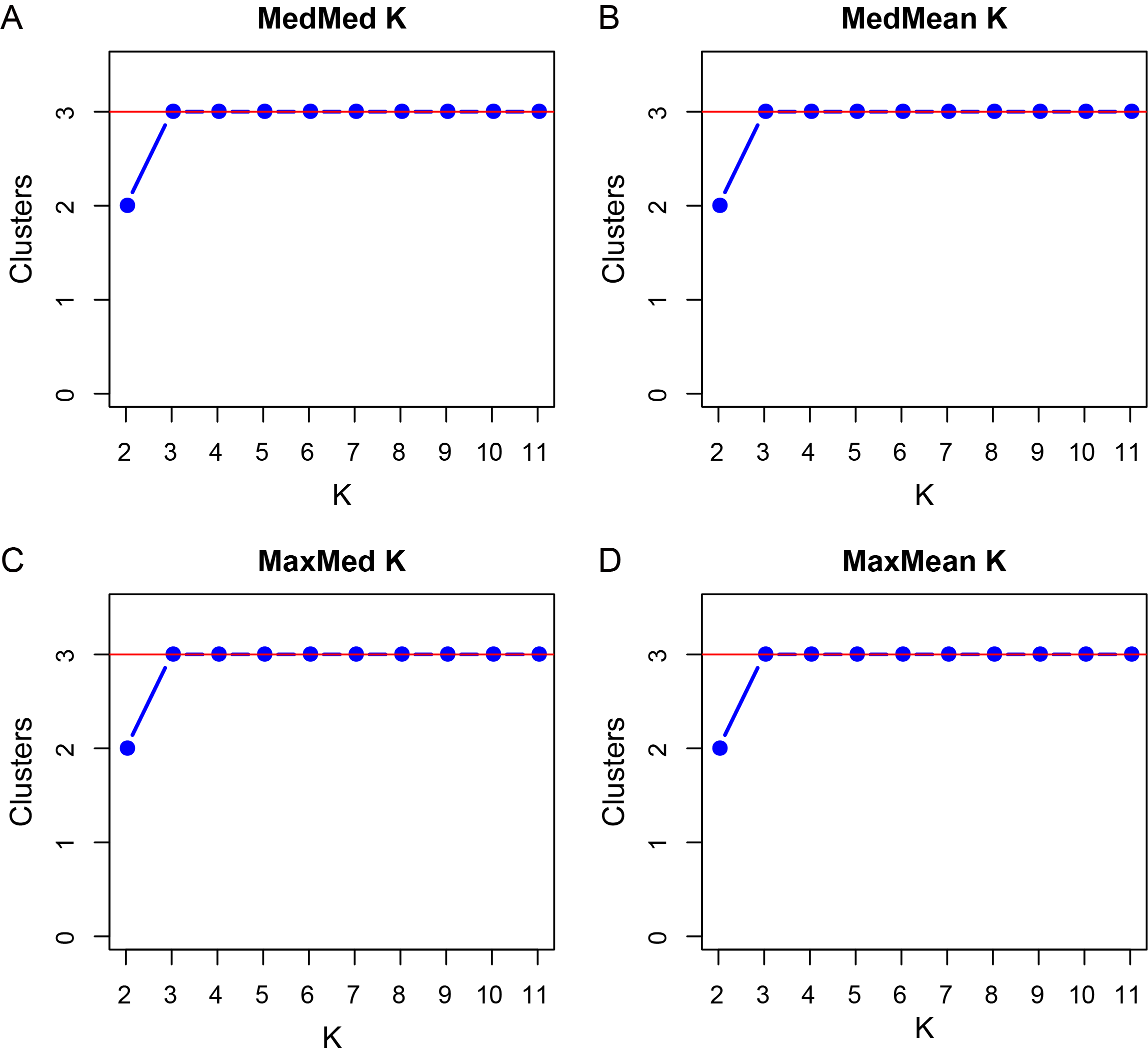


**Supplementary Figure 2.** Marginal likelihood value in different K of fastSTRUCTURE analysis.
